# Assessing Workplace Relational Civility (WRC) with a New Multidimensional “Mirror” Measure

**DOI:** 10.3389/fpsyg.2016.00890

**Published:** 2016-06-20

**Authors:** Annamaria Di Fabio, Alessio Gori

**Affiliations:** Department of Education and Psychology (Psychology Section), University of FlorenceFlorence, Italy

**Keywords:** workplace civility, decent work, relationships and work, relational civility, relational theory, relational decency, relational culture, relational readiness

## Abstract

This article first introduces a new psychological construct and then presents the psychometric properties of a new multidimensional measure for assessing workplace relational civility (WRC). This new self-report mirror measure has two specular sections (Part A—Me with others; Part B—Others with me) that can assess individuals' relational patterns regarding civility and that can help reduce the bias in the assessment process. The results of exploratory factor analysis revealed a factor structure with three robust dimensions [relational decency (RD), relational culture (RCu), and relational readiness (RR)] exhibiting good values of internal consistency. Confirmatory factor analysis showed, in turn, a good fit of the model to the data. The correlations between the sections of the measure and the 11 instruments used were significant and in the expected directions. These results suggest that this new, brief mirror measure for assessing WRC can be easily employed in different organizational contexts to open different typologies of actions on the basis of the three dimensions, thereby facilitating the construction of a safer and more decent relational work environment.

## Introduction

Positive psychology is being used more frequently these days to improve the quality of work life and organizational effectiveness. Seligman and Csikszentmihalyi (2000) maintain that the purpose of positive psychology is “to begin to catalyze a change in the focus of psychology from pre-occupation only with repairing the worst things in life to also building positive qualities” (Seligman and Csikszentmihalyi, 2000, p. 5). Positive psychology aims at helping individuals develop their potential and make the best choices for their personal and professional lives (Seligman, 2002; Ryff and Singer, 2008; Waterman et al., 2010; Savickas, 2011; Guichard, 2013).

In other words, positive psychology helps individuals develop effective and lifelong self and relational management (Di Fabio, 2015a) across the numerous personal and professional transitions and complex challenges of the 21st century (Blustein, 2011; Di Fabio and Maree, 2013; Di Fabio, 2014a; Di Fabio and Kenny, 2015).

This effective and lifelong self and relational management (Di Fabio and Kenny, 2016) can impact directly and indirectly on the sphere of professional life and help develop and improve psychological resources and talents. It can also promote opportunities for women and men to obtain decent and productive work in conditions of freedom, equality, security, and human dignity (ILO, 1999). According to the United Nations (1948), everyone who works has the right to just and favorable remuneration ensuring for himself and his family an existence worthy of human dignity, and supplemented, if necessary, by other means of social protection (United Nations, 1948). The concept of decent work is thus characterized by dignity and decency in the workplace, which are part and parcel of equality of opportunity, social protection, social security, and quality of work (Faioli, 2009). Quality of work, in particular, is the basis of decent work and can be linked to the quality of relationships in the workplace; in other words, decent work is linked to positive relational outcomes (Blustein et al., 2016; Di Fabio and Kenny, 2016; Kenny et al., 2016). It then follows that quality of work and quality of relationships influence each other. In fact, some authors see work and relationships as major social constructs (Blustein, 2011; Richardson, 2012).

The relational theory of working (Blustein, 2011) provides a framework for understanding how working is embedded in external and internal relational contexts and how it can be viewed as an inherently relational act. Thus, on the one hand, the psychology-of-working framework seeks to explore the impact of intrapsychic, relational, social, economic, political, and historical factors on people's work lives, and, on the other hand, it seeks to identify three human needs that working optimally can fulfill: the need for survival and power, the need for social connection, and the need for self-determination (Blustein, 2006, 2011). The theory also emphasizes the importance of creating optimal conditions for the development of adaptive relationships among peers and supervisors at work (Blustein, 2011): relationships that need to be characterized by civility.

Traditionally, the work and organizational literature has focused on workplace incivility (WI) rather than civility at work. Andersson and Pearson (1999) define WI as “low intensity deviant behavior with ambiguous intent to harm the target, in violation of workplace norms of mutual respect. Uncivil behaviors are characteristically rude and discourteous, displaying a lack of regard for others” (p. 457). Cortina et al. (2001) refer to it as a form of interpersonal mistreatment in the workplace characterized by violence, aggression, bullying, tyranny, harassment, deviance, and injustice (Cortina et al., 2001). Other authors talk about physical forms of aggression (VandenBos and Bulatao, 1996; Griffin et al., 1998; Leather et al., 1999) or psychological forms of aggression that intentionally cause injury (Baron and Neuman, 1996; Folger and Baron, 1996; Neuman and Baron, 1997; Glomb, 1998).

This article seeks to go beyond the conceptualization of incivility at work and to focus rather on the utility of models that include positivity and early interventions as forms of prevention (Hage et al., 2007; Kenny and Hage, 2009). These are more effective when efforts to increase resources and competencies (Boyatzis et al., 2002, 2015a; Di Fabio, 2006; Boyatzis and Saatcioglu, 2008; Di Fabio and Bernaud, 2008; Boyatzis, 2009; Di Fabio and Palazzeschi, 2012, 2015a, 2016a; Di Fabio et al., 2013; Di Fabio and Saklofske, 2014a,b; Di Fabio and Bucci, 2015) are combined with efforts to decrease risks, in line with recent advances reported in the positive psychology literature (Di Fabio and Kenny, 2012a,b; Kenny et al., 2014). So, given the importance of the concepts of relationality and civility, we move from the idea of decent work and well-being at work—through relationality—to the idea of respect and caring for the self and others and the relationships between people (Blustein, 2006; Di Fabio and Maree, 2012, 2016; Maree, 2013; Di Fabio, 2015b; Di Fabio and Bucci, 2016a). To this end, we developed a new construct: the relational civility (RC) construct.

## Relational civility: construct definition

Civility has been defined in different ways, but almost all definitions state that this construct is characterized by respect and courtesy and a general awareness of the rights of others (Elias, 1982; Carter, 1998).

The *Random House Dictionary* defines civility as “courtesy and politeness toward fellow human beings” (2015). Civility has also been defined as a source of power, a means of gaining favor and asserting cultural superiority, attaining social advantage (Elias, 1982; Morris, 1996), and “the sum of the many sacrifices we are called to make for the sake of living together” (Carter, 1998, p. 11). Some researchers stress the virtue aspects of the moral implications of civility (Elias, 1982; Carter, 1998) while others emphasize the “way of signaling the existence of self-control” in civility (Wilson, 1993, p. 83). Most researchers agree, however, that the need for civility becomes greater when the interaction among people increases in complexity and frequency (Goffman, 1967; Elias, 1982; Chen and Eastman, 1997; Carter, 1998). To paraphrase Blustein (2006), we could say that civility is intrinsically relational.

We developed the concept of RC on these premises as a form of relational style characterized by respect and concern for the self and others, interpersonal sensitivity, personal education, and kindness toward others. RC also includes civil behaviors such as treating others with dignity and respecting social norms to facilitate peaceful and productive cohabitation.

RC underlines the importance of interpersonal behavior and is based on the assumption that people with their own expectations, cognitive schemas, and cultural backgrounds interact and influence each other. In other words, RC is a dynamic concept that can be adapted to different contexts. For example, we can refer to RC at work, RC in sport, RC in academia, and so on. This construct can thus be adapted to many spheres in our lives.

The definition of the construct covers many factors related to civility and many factors related to relationality which can be grouped into three principal dimensions: relational decency (RD), relational culture (RCu), relational readiness (RR).

RD, a new concept in the psychology literature, is inspired by positive psychology (Fredrickson and Losada, 2005) and concerns optimal relational functioning characterized by decency in relationships, respect for the self and for others, being able to express opinions freely, being assertive, and being tactful. Having a high level of RD means being able to understand the relational dynamics in a situation and integrating them in order to devise and promote constructive relationships. In other words, RD enables individuals to have positive and decency-based relationships characterized by assertiveness, freedom to express ideas, respect for others, and tact in sustaining convictions. RD can thus contribute to relational well-being and happiness.

RCu, another new concept in the psychology literature, is inspired by relational cultural theory (Miller, 1976; Miller et al., 2004). It concerns the complexity of human relationships in terms of connection and disconnection and also recognizes and explores the social implications of psychology theory and the influence of culture on the quality and nature of relationships and the subsequent effect on healthy coexistence (McCauley, 2013). RCu thus comes from a distance and can vary across cultures. People with good RCu are able to communicate with kindness and deal with others in a polite manner through effective diversity management (Harris et al., 2013; Di Fabio, 2016b).

RR, a further new concept in the psychology literature concerns speed in understanding the feelings of others and showing proactive sensibility. People with good IR are thus able to read the emotions of others easily and to demonstrate delicacy, empathy, compassion, and attention to their reactions.

RC can be seen as a collection of relational factors that are central to developing satisfactory relationships with others and promoting well-being among people.

The need for greater civility is evident when interactions among people become more complex requiring them to attune their behavior to that of others (Andersson and Pearson, 1999) in order to prevent misunderstandings (Elias, 1982) and to promote well-being (Boyatzis, 2009). In line with these premises, the present study aimed at presenting a much-needed new multidimensional measure to assess workplace relational civility (WRC).

### Differences and similarities with similar constructs

When developing a new construct it is essential to compare it—in this case WRC—with other similar constructs in order to identify and analyze the similarities and the differences.

In the field of work and organizational psychology, constructs similar to WRC are organizational citizenship behavior (OCB) and prosocial organizational behavior (POB). First of all, we can speculate that WRC is a prerequisite for prosocial and citizenship behaviors; in fact, when there is a high level of WRC, it can be assumed that there will also be high levels of prosocial and citizenship behaviors. Because WRC precedes these other two constructs and is necessary for the development of healthy attachments, some specific differences must exist.

OCB is defined as “an individual behavior that is discretionary, not directly or explicitly recognized by the formal reward system, and that in the aggregate promotes the effective functioning of the organization” (Organ, 1988, p. 4). Here, the variables that may most frequently overlap between WRC and OCB are the courtesy, the altruism, and the civic virtues.

Regarding the courtesy virtue of the OCB, emphasis is placed on keeping colleagues and superiors informed about organizational matters and working conditions. The courtesy virtue of the WRC, on the other hand, seems to be broader and to concern a general lifestyle and consequently a way of being, a relational modality geared to education, kindness, and courtesy in the workplace. The courtesy of the WRC may thus subsume the courtesy of the OCB.

There could also be similarities between the RCu factor of the WRC and the altruism factor of the OCB. RCu is a relational style that is polite, kind, and courteous, and because of the high level of education and politeness of people with a high level of RCu, it is generally easy for them to behave altruistically. In OCB, the altruism factor is restricted to helping people in their work activities whereas in RCu, it is more specifically focused on being comfortable with and recognizing other people as human beings. Civic virtue (CV), defined as having an interest in the organization as a whole and a willingness to participate actively in its governance, may have links with some aspects of RR, particularly regarding interest in others. CV can also be linked to RCu in respect of the subcategory of level of education, which, in turn, is linked to CV.

POB, defined as the behavior of a member of an organization who provides services to co-workers, customers, teams, or the organization itself (McNeely and Meglino, 1994) on a voluntary basis (Clary et al., 1998), can be considered part of civility at work. However, POB seems to refer to relevant aspects of work and not necessarily to relationships in general, unlike WRC. Also, the self-improvement aspect of POB can be linked to some aspects of WRC, particularly in relation to the evolution of the self; but POB does not include the ability to express opinions and ideals and a decency-based relationship at work, which are aspects of WRC.

WRC goes beyond the concepts of citizenship and prosocial behaviors as it is a dynamic concept that combines a sense of relationality (Blustein, 2011) and respectivity (Maree, 2012) in civility at work (Di Fabio, 2015b). WRC refers to behavior in the workplace that is decent, prosocial, polite, careful, and that involves relational style patterns that are in line with one's involvement with others. In other words, we could say that WRC is positioned at the meta-level compared to these above constructs.

## Workplace relational civility (WRC): scale development

In developing the WRC construct, we considered the specific components of workplace behavior discussed earlier.

These components are:

1. Relational decency (RD) at work (decency in relationships, respect for the self and others, assertiveness, ability to express convictions, relational capacity);
2. Relational culture (RCu) at work (politeness, kindness, high level of education, courteousness);
3. Relational readiness (RR) at work (sensibility towards others, ability to read the emotions of others, concern for others, delicacy, empathy, compassion, and attention to the reactions of others).

In devising the process of measurement, we selected various items for each subcategory of our theoretical model. The response format adopted was a Likert scale with five answer options (1 = not at all; 2 = a little; 3 = somewhat; 4 = a lot; 5 = a great deal).

Based on the assumption that WRC is relational, we developed a new “mirror” kind of measurement that assesses a concept from one's own perspective and, at the same time, assesses the same concept from the perspective of others. In other words, the participants in the study were first asked to describe their relationship with others over the past 3 months, and then to respond to statements, with the same content, that describe the relationship of others with them over the same time period (the past 3 months). This kind of measurement enables a better assessment of the interpersonal patterns and identifies the discrepancies between how the subject looks at himself or herself during the interaction with others and how the subject views the other persons in their interaction with him or her. This “mirror” measurement can lead to a more balanced evaluation by helping to reduce bias in the assessment process. This kind of measurement can place a person in a position to reflect on his or her own actions and to analyze the behavior of others, thereby making him or her more aware of the relational dynamics.

A measure consisting of two specular parts (A and B) was developed. In the first phase of the study, we tested the factor structure of the preliminary version of the WRC scale. A convenience sample consisting of 80 students (60 women, 20 men) enrolled in various psychology courses at the University of Florence and 26 employees (12 women, 14 men) completed this preliminary version. Exploratory factor analysis (EFA) was used to verify the factor structure of the scale. Velicer's minimum average partial (MAP) criterion and the inspection of the scree plot suggested a three-factor solution for the two parts (A and B) of the measure. In order to obtain a clear, robust factor solution, and on the basis of the factor analysis criteria, we decided to eliminate the items with communalities under 0.30 and reached a version with 13 specular items for each part.

## Methods

### Participants and procedure

The participants in the study were 115 workers (79.1% male, 20.9% female) with a mean age of 43.96 years (*SD*, 12.74). The participants first completed the WRC scale and then, in order to assess some aspects of the concurrent validity of this new mirror measure, filled in 11 instruments that assessed similar as well as different constructs. All the instruments were administered in accordance with the norms regarding the privacy and anonymity of participants.

Written informed consent was received from each of participants after a full description of the study. The participants were told also that they were free to withdraw from the study at any time and that there would be no payment for participating. With regard to ethical standards for research, the study adhered to the latest version of the Declaration of Helsinki revised in Fortaleza [World Medical Association (WMA), 2013].

### Measures

#### Workplace relational civility (WRC)

The WRC measure is a self-report mirror instrument of 26 items that assesses RC at work. The WRC measure has three dimensions: relational readiness (RR) at work, RCu at work, and RD at work. The sum of these dimensions gives an overall score for WRC for each part (Part A and Part B). The response format adopted was a Likert scale with five responses (1 = not at all; 2 = a little; 3 = somewhat; 4 = a lot; 5 = a great deal) (see Appendix).

#### Organizational citizenship behavior (OCB; Podsakoff et al., 1990)

The OCBs measure is an instrument that assesses the dimensions of civic virtue defined as “behavior on the part of an individual that indicates that he/she responsibly participates in, is involved in, or is concerned about the life of the company” (Podsakoff et al., 1990, p. 115). Podsakoff et al. (1990) developed a 24-item instrument with five dimensions of OCB: altruism, conscientiousness, sportsmanship, courtesy, and civic virtue. Examples of these items: altruism “Helps others who have heavy work load”; conscientiousness “Attendance to work is above the norm”; sportsmanship “Always focuses on what's wrong, rather than the positive side”; courtesy “Takes steps to try to prevent problems with other workers,” and civic virtue “Attends functions that are not required, but help the company's image.” A seven-point scale was used in the OCB with response categories ranging from one (strongly disagree) to seven (strongly agree). In this study, we used the Italian version of the measure (Di Fabio and Palazzeschi, 2016b), which showed good values of internal consistency (altruism = 0.84; conscientiousness = 0.81; sportsmanship = 0.84; courtesy = 0.83; civic virtue = 0.80).

#### Prosocial organizational behaviors (POB; McNeely and Meglino, 1994)

McNeely and Meglino (1994) developed a measure of POB asking respondents to classify items into one of the three categories of prosocial behavior. Based on this process, they selected the 20 items with the lowest level of ambiguity regarding the classification (McNeely and Meglino, 1994). These 20 items, used to evaluate prosocial behavior, were grouped into three main factors: Factor 1 = prosocial organizational behavior (e.g., “Speaks favorably about the organization to outsiders”); Factor 2 = role-prescribed prosocial behavior (e.g., “Arrives at work on time”); Factor 3 = prosocial individual behavior (e.g., “Assists co-workers or students with personal problems.”). A five-point scale was used with response categories ranging from 1 = never to 5 = always. The psychometric properties of the Italian version of this measure used in this study were good (prosocial organizational behavior = 0.81; role-prescribed prosocial behavior = 0.84; prosocial individual behavior = 0.83; Di Fabio and Bucci, 2016b).

#### Intrapreneurial self-capital scale (ISCS; Di Fabio, 2014b)

Intrapreneurial self-capital is defined as a core of individual intrapreneurial resources that are used to cope with career and life construction challenges and that include dimensions of core self-evaluation, hardiness, creative self-efficacy, resilience, goal mastery, decisiveness, and vigilance (Di Fabio, 2014b). The Intrapreneurial Self-Capital Scale (ISCS) was developed by Di Fabio (2014b) to measure the intrapreneurial self-capital construct. The ISCS uses a five-point Likert-type scale (1 = strongly disagree, 2 = disagree, 3 = neither agree nor disagree, 4 = agree, 5 = strongly agree) and consists of 28 items (e.g., “I am able to deal with most of my problems,” “I'm able to improve the ideas produced by others,” “I'm able to achieve objectives despite obstacles,” “One of my goals in training is to learn as much as I can”). In the present study, we used the Italian version of this measure, which showed good internal consistency (*a* = 0.86; Di Fabio, 2014b).

#### Psychological self-capital questionnaire (PSQ; Luthans et al., 2007)

Psychological capital is a new construct measured with the Psychological Capital Questionnaire (PCQ). This questionnaire consisting of 24 items has four subscales: (1) hope (e.g., “I feel confident contributing to discussions about the organization's management”), (2) optimism (e.g., “I can think of many ways to reach my current work goals”), (3) optimism (e.g., “I'm optimistic about what will happen to me in the future regarding work”), and (4) resilience (e.g., “I usually take stressful things at work in my stride”). Each of these subscales has six items with response options on a six-point Likert scale ranging from one (strongly disagree) to six (strongly agree). The PCQ has good psychometric properties also in the Italian version that was used in the present study: (hope = 0.75; efficacy = 0.78; resilience = 0.70; optimism = 0.77; the overall scale = 0.81; Alessandri et al., 2015).

#### Flourishing scale (FS; Diener et al., 2010)

The Flourishing Scale is a self-report measure consisting of eight items that assesses respondents' self-perceived success in important areas of their lives such as relationships, self-esteem, purpose, and optimism. Examples of the items: “My social relationships are supportive and rewarding,” “I lead a purposeful and meaningful life,” “I am optimistic about my future.” This scale provides a single total score (Diener et al., 2009). In this study, the Italian version of the scale was used (*a* = 0.88; Di Fabio, 2016a).

#### Workplace incivility scale (WIS; Cortina et al., 2001)

The Workplace Incivility Scale (WIS) is a seven-item scale that assesses the frequency of respondents' experiences of disrespectful, rude, or condescending behavior from supervisors or co-workers during the past five years (Cortina et al., 2001). Examples of the items: “Put you down or was condescending to you,” “Doubted your judgment on matters over which you have responsibility,” and “Paid little attention to or showed little interest in your opinion.”

The response format is a five-point Likert scale ranging from one (never) to five (often). In this study, the Italian version of the scale was used (*a* = 0.91; Di Fabio and Ghizzani, 2010).

#### Positive relational management scale (PRMS; Di Fabio, 2015b)

The Positive Relational Management Scale (PRMS) is a measure consisting of 12 items with responses on a five-point Likert scale ranging from one (strongly disagree) to five (strongly agree). This scale assesses three dimensions (respect, caring, connection) and gives a total score. Examples of the items: respect: “I keep a balance between respect for others and for myself”; caring: “I often take care of others”; connection: “I have good relationships with my family”). The psychometric properties of the scale are good also in its Italian version. (respect = 0.81; caring = 0.79; connection = 0.80; PRMS total = 0.84; Di Fabio, 2015b).

#### Satisfaction with life scale (SWLS; Diener et al., 1985)

The Satisfaction With Life Scale (SWLS) is a self-report instrument that measures global life satisfaction. It consists of five items with responses on a seven-point Likert scale with higher values corresponding to a higher degree of life satisfaction. Examples of the items: “I am satisfied with my life,” “The conditions of my life are excellent.” The psychometric properties of the SWLS are good, with different studies reporting a unidimensional structure of the measure (Diener et al., 1985; Vera-Villarroel et al., 2012). In this study, the Italian version of the scale was used (*a* = 0.85; Di Fabio and Gori, 2015).

#### Rosenberg self-esteem scale (RSES; Rosenberg, 1965)

The Rosenberg Self-Esteem Scale (RSES) is a 10-item scale for assessing global self-esteem with the items answered on a four-point Likert scale ranging from strongly agree to strongly disagree. Examples of the items: “On the whole, I am satisfied with myself,” “I have a positive attitude toward myself.” The psychometric properties of the RSES have been reported as good in several studies (Corwyn, 2000). In this study, the Italian version of the scale was used (*a* = 0.84; Prezza et al., 1997).

#### Multidimensional scale of perceived social support (MSPSS; Zimet et al., 1988)

The Multidimensional Scale of Perceived Social Support (MSPSS) is a 12-item scale for assessing perceived social support with the items answered on a seven-point Likert-type scale ranging from strongly disagree to strongly agree that measures perceived support in three main domains: family, friends, and a significant other. Examples of the items: “My family works very hard to help me,” “I can speak about my problems with my friends,” “When I need someone, there is always a special person who stands by me.” Higher scores indicate higher levels of perceived social support. The psychometric properties of the MSPSS have been reported as good in several studies with different samples (Zimet et al., 1988, 1990). In the present study, the Italian version of the scale was used and reported excellent internal consistency for the three factors: family (α = 0.91), friends (α = 0.93), and a significant other (α = 0.88), and the total score (α = 0.92; Di Fabio and Palazzeschi, 2015b).

#### Trait emotional intelligence questionnaire—short-form (TEIQue-SF; Petrides and Furnham, 2006; Cooper and Petrides, 2010)

The Trait Emotional Intelligence Questionnaire (TEIQue) is a 153-item self-report measure that measures 15 dimensions, four factors, and a global trait for EI. The Trait Emotional Intelligence Questionnaire—Short form (TEIQue-SF) is a 30-item questionnaire also designed to measure global trait emotional intelligence (trait EI). It is based on the full form of the TEIQue. Two items from each of the 15 dimensions of the TEIQue were selected for inclusion, based primarily on their correlations with the corresponding total dimension scores (Petrides and Furnham, 2006; Cooper and Petrides, 2010). The questionnaire provides a total score as well as scores for four principal dimensions: well-being (e.g., “I feel that I have a number of good qualities,” “On the whole, I'm pleased with my life”), self-control (e.g., “I'm usually able to calm down quickly after I've got mad at someone,” “I would describe myself as a calm person”), emotionality (e.g., “I often find it difficult to recognize what emotion I'm feeling,” “I find it difficult to tell others that I love them even when I want to”), and sociability (e.g., “I can deal effectively with people,” “If I wanted to, it would be easy for me to make someone angry”). The Italian version of the scale has been shown to have good internal consistency coefficients for well-being (α = 0.83), sociability (α = 0.84), self-control (α = 0.81), emotionality (α = 0.82), and for the total score (α = 0.85; Di Fabio and Palazzeschi, 2011).

### Data analysis

Factor analysis was used to identify attachment dimensions, with the objective of assessing the validity of the hypothesized construct (WRC). A series of exploratory factor analyses (EFAs) together with maximum likelihood (ML) analyses were conducted to verify the factor structure of the WRC.

As suggested in most of the relevant literature, the number of components extracted was based on the percentage of variance accounted for, the Kaiser-Guttman method, and the scree plot (Nunnally and Bernstein, 1994; Giannini et al., 2014).

We then performed a confirmatory factor analysis (CFA), and, to evaluate the model's goodness of fit, a number of indexes were used. Since the chi-square fit index depends on sample size (Schermelleh-Engel et al., 2003), two relative fit indexes were considered because they can generally be used in large as well as small samples: the TLI (Tucker-Lewis Index) and the CFI (Comparative Fit Index; Bentler, 1990). Values of these indexes higher than 0.90 indicate satisfactory fit. In addition, the SRMR (Standardized Root Mean Square Residual) and the RMSEA (Root Mean Square Error of Approximation) were used because they are currently two of the most popular measures of model fit and provide fundamental indications of how well a proposed theory fits the data (Hooper et al., 2008; Giannini et al., 2011; Craparo et al., 2015; Di Fabio and Gori, 2015; Gori et al., 2015). Reliability for each scale was calculated using Cronbach's alpha coefficient (Cronbach, 1951), several aspects of concurrent validity were verified using Pearson's *r* coefficient, and statistical analyses were conducted using SPSS 18.0 and AMOS 6.0.

## Results

### Construct validity

Examination of the scree plot (Cattell, 1966), the percentage of variance accounted for, and the structure matrix (Horn, 1965; Zwick and Velicer, 1986; Glorfeld, 1995) indicated that as many as three factors should be retained for rotation. The results of the exploratory factor analysis (EFA) (Promax rotation) showed a factor structure with three principal dimensions for both Part A and Part B of the WRC construct. So, on the basis of the factor analysis criteria, we extracted three factors accounting for 60.44% of the total variance for Part A (eigen-values > 1; 5.26, 1.51, 1,10), and, likewise, we extracted three factors accounting for 71.22% of the total variance for Part B (eigen-values > 1; 6.74, 1.37, 1.15). The factor structure matrix shows the three independent and specular factors of the two parts of the WRC construct (see Table 1).

**Table 1:** **Factor structure matrix for the two parts of WRC**.

**Items**	**WRC part A**			**WRC part B**			
	**Relational readiness**	**Relational culture**	**Relational decency**	**Items**	**Relational readiness**	**Relational culture**	**Relational decency**
12	0.955			10	0.905		
13	0.823			12	0.840		
10	0.794			13	0.730		
9	0.393			9	0.624		
11	0.353			11	0.488		
6		0.932		6		0.898	
7		0.787		5		0.769	
5		0.472		7		0.753	
8		0.445		8		0.534	
2			0.806	1			0.829
1			0.651	4			0.726
4			0.650	2			0.714
3			0.580	3			0.545

In order to verify the factor structure, we performed a confirmatory factor analysis (CFA) on the basis of the EFA results that indicated three main factors. The goodness-of-fit indexes showed an excellent fit of the model to the data for Part A (see Table 2). As regards Part B, although the chi-square was significant (*p* < 0.001), and the Tucker-Lewis Index (TLI) and the Comparative-Fit Index (CFI) showed acceptable values and confirmed the three-factor solution for Part B of the WRC, the value of the root mean error of approximation (RSMEA) indicated poor fit (see Table 2).

**Table 2:** **Summary of CFA fit indices for WRC (Part A and Part B)**.

**Sample (*n* = 115)**	**χ2/*df***	**TLI**	**CFI**	**SRMR**	**RMSEA**
WRC (Part A)	1.14, *p* = 0.210	0.977	0.982	0.033	0.038
WRC (Part B)	1.93, *p* = 0.001	0.915	0.932	0.046	0.096

TLI, Tucker-Lewis index; CFI, Comparative Fit Index; SRMR, Standardized Root Mean Square Residual; RMSEA, Root Mean Square Error of Approximation.

### Reliability

The internal consistency of the WRC scale, calculated using Cronbach's alpha coefficient, showed excellent internal coherence of the instrument for the total score of both Part A and Part B of the WRC (Part A, *a* = 0.87; Part B, *a* = 0.92). Cronbach's alphas for the three factors extracted for Part A were: Factor 1A = relational readiness (*a* = 0.83); Factor 2A = relational culture (*a* = 0.76); Factor 3A = relational decency (*a* = 0.75); while for Part B they were: Factor 1B = relational readiness (*a* = 0.86); Factor 2B = relational culture (*a* = 0.88); Factor 3B = relational decency (*a* = 0.85). Calculations of item-total correlation indicated that all the items correlated significantly and in a positive direction with the total score of both Part A and Part B of the WRC, with the correlations ranging from a minimum of 0.48 to a maximum of 0.78 for Part A and from a minimum of 0.55 to a maximum of 0.81 for Part B.

### Convergent and divergent validity

In the present study, WRC showed strong correlations with the Italian version of the following measures: the OCBs scale, the POBs scale, the flourishing scale, the positive relational management scale, the intrapreneurial self-capital scale, and the psychological self-capital scale. Correlations between WRC and these instruments were positive and statistically significant, attesting to a good convergent validity (see Table 3). Also, correlations between WRC and the other instruments described in the Measures Section of this article were in the expected directions and showed a good divergent validity of the WRC construct (see Tables 4, 5).

**Table 3:** **Summary of correlations among the two parts of WRC, OCB, and POB factors**.

	**Organizational Citizenship Behavior (OCB)**					**Prosocial Organizational Behavior (POB)**		
	**Conscientiousness**	**Sportsmanship**	**Civic virtue**	**Courtesy**	**Altruism**	**POB**	**RPPB**	**PIB**
Relational readiness	0.257^**^	−0.036	0.295^**^	0.216^*^	0.349^**^	0.312^**^	0.256^**^	0.194^*^
Relational culture	0.248^**^	0.124	0.213^*^	0.248^**^	0.298^**^	0.131	0.214^*^	0.014
Relational decency	0.317^**^	0.149	0.340^**^	0.378^**^	0.436^**^	0.312^**^	0.290^**^	0.244^**^
WRC part A	0.332^**^	0.082	0.345^**^	0.333^**^	0.438^**^	0.313^**^	0.309^*^	0.188^*^
Relational readiness	0.177	−0.062	0.151	0.131	0.173	0.389^**^	0.327^**^	0.289^**^
Relational culture	0.139	0.105	0.169	0.233^*^	0.203^*^	0.336^**^	0.380^**^	0.113
Relational decency	0.159	0.014	0.051	0.113	0.120	0.315^**^	0.314^**^	0.133
WRC part B	0.184^*^	0.017	0.144	0.181	0.191^*^	0.402^**^	0.392^**^	0.211^*^

** p < 0.01;

* p < 0.05. POB, Prosocial Organizational Behavior; RPPB, Role-Prescribed Prosocial Behavior; PIB, Prosocial Individual Behavior.

**Table 4:** **Summary of correlations among the two parts of WRC, ISC, PSC, F, TEIQue-SF, PSR**.

	**Intrapreneurial self-capital (ISC)**	**Psychological self-capital (PSC)**	**Flourishing (F)**	**Trait emotional intelligence questionnaire—SF**	**Positive relational management (PRM)**
Relational readiness	0.450^**^	0.564^**^	0.403^**^	0.284^**^	0.372^**^
Relational culture	0.433^**^	0.452^**^	0.430^**^	0.289^**^	0.352^**^
Relational decency	0.440^**^	0.476^**^	0.512^**^	0.439^**^	0.435^**^
WRC part A	0.538^**^	0.614^**^	0.540^**^	0.403^**^	0.469^**^
Interpersonal readiness	0.305^**^	0.397^**^	0.351^**^	0.166	0.320^**^
Relational culture	0.465^**^	0.356^**^	0.420^**^	0.278^**^	0.330^**^
Relational decency	0.315^**^	0.334^**^	0.383^**^	0.193^*^	0.300^**^
WRC part B	0.413^**^	0.420^**^	0.442^**^	0.242^**^	0.365^**^

** p < 0.01;

* p < 0.05.

**Table 5:** **Summary of correlations among the two parts of WRC, SWLS, RSES, MSPSS, WI**.

	**SWLS**	**RSES**	**MSPSS**	**WI**
Relational readiness	0.299^**^	0.106	0.311^**^	–0.020
Relational culture	0.344^**^	0.230^*^	0.308^**^	–0.054
Relational decency	0.407^**^	0.204^*^	0.319^**^	–0.036
WRC part A	0.420^**^	0.210^*^	0.381^**^	–0.042
Interpersonal readiness	0.337^**^	0.013	0.202^*^	0.032
Relational culture	0.344^**^	0.175	0.137	–0.131
Relational decency	0.336^**^	0.178	0.147	0.038
WRC part B	0.391^**^	0.135	0.189^*^	–0.020

** p < 0.01;

* p < 0.05. SWLS, Satisfaction with Life Scale; RSES, Rosenberg Self-Esteem Scale; MSPSS, Multidimensional Scale of Perceived Social Support; WI, Workplace incivility.

## Discussion

The aim of this article was to introduce the novel multidimensional construct of WRC on how individuals interface with and act toward others in the workplace, and also the construct's mode of assessment. Thanks to this mirror assessment procedure, people can describe how they used to relate to each other, and, at the same time, how others related to them in a work environment. This assessment procedure helps subjects reduce assessment bias and better evaluate their interpersonal style by identifying the discrepancies between how they look at themselves during interactions with others and how they view others during their interactions with them. This new way of measurement promotes the growth of an awareness process through the analysis of three main dimensions conceptualized as relational readiness (RR), RCu, and RD. These dimensions are globally related to an optimal range of relational functioning that identifies a level of growth and relational capacity that is characterized by decency in relationships, respect for the self and others, being able to express opinions freely, assertiveness, good taste, empathy, compassion, and awareness of the effect that their words can have on others. People with these characteristics are generally able to communicate with others with kindness and to attune their expressions and behaviors to those of others to promote a climate of mutual respect. WRC is thus important because it can promote a climate of decency, respect, and awareness between people, thereby inspiring others through civility (Boyatzis and McKee, 2006).

The results of this study showed good psychometric properties for the WRC instrument. In order to verify its dimensional structure, we carried out confirmatory factor analysis through structural equation modeling. On the basis of the results of the EFA and the CFA, the final model consisted of three independent, robust factors with a good fitting model for the two parts of the instrument. In particular, the results showed that the SRMS and the RMSEA were generally below 0.05, except for the RMSEA value for Part B (i.e., 0.096). Regarding this RMSEA result, while some researchers (Browne and Cudeck, 1993; MacCallum et al., 1996) have used 0.01, 0.05, and 0.08 to indicate excellent, good and mediocre fit respectively, other researchers consider 0.10 as a reasonable cutoff for good versus poor fitting models if the other indexes used to verify the model fit are good or acceptable, which was the case in this study (Kenny et al., 2015). All the fit indexes indicated the goodness of the hypothesized model (Hu and Bentler, 1999) thus confirming the three-dimensional factorial structure. Reliability analysis showed values ranging from acceptable to excellent for all the factors in line with the following rules of George and Mallery (2003): “α > 0.9 = Excellent, α > 0.8 = Good, α > 0.7 = Acceptable, α > 0.6 = Questionable, α > 0.5 = Poor, and α > 0.5 = Unacceptable.” The minimum value of alpha was for the Factor 3A = relational decency (*a* = 0.75), and even it was in the range between acceptable and good, also bearing in mind the restricted number of items for each factor.

Correlations between the two parts of the WRC construct and the measures used to verify some aspects of concurrent validity showed good values: all the relationships among the variables under investigation were in the right direction with the right significance.

In line with these results and in the spirit of positive psychology, we should talk about WRC rather than WI in order to promote effective and lifelong self and relational management (Di Fabio, 2015a) across the numerous personal and professional transitions and complex challenges of the 21st century (Blustein, 2011; Di Fabio and Maree, 2013; Di Fabio, 2014a; Di Fabio and Gori, 2016). Indeed, focusing on WRC gives significant value to the preventive approach in psychology (Hage et al., 2007; Kenny and Hage, 2009), specifically in endeavoring to increase resources and, at the same time, decrease risks. This new brief, mirror measure (WRC) thus promotes positive behavior in a framework of primary prevention (Hage et al., 2007; Kenny and Hage, 2009; Di Fabio and Kenny, 2015; Di Fabio et al., in press), and helps build a relational work environment that is safer and more decent.

## Limitations and future directions

The study had a number of shortcomings: first of all, the number of participants was small because of the difficulty of recruiting subjects with the required features. Future studies should therefore examine this novel construct (WRC) and its mirror measurement assessment using a bigger sample. It would also be interesting to examine the construct and the assessment in a cross-cultural context in order to investigate the role of the complexity of human relationships from a cultural point of view. Future research should also examine WRC in relation to other promising variables in organizational contexts such as ability-based emotional intelligence and trait emotional intelligence as well as other variables such as positive affect for hedonic well-being, meaning in life for eudaimonic well-being and also competency based perspective (Boyatzis et al., 2002, 2015a,b; Boyatzis and Saatcioglu, 2008; Boyatzis, 2009; Hazy and Boyatzis, 2015). WRC could be also studied in relation to objective outcomes of performance in organizational contexts.

However, on the basis of the results of the present study, we can affirm that the WRC scale is a brief mirror measure with good psychometric properties that can promote individuals' strengths and also the growth of decent work.

## Author contributions

AD conceptualized the study and choose the theoretical framework. AD and AG conceptualized the new scale and realized it. AD and AG collected the data. AG analyzed the data and wrote the methods and results. Then all authors wrote the paper together and read and revised the manuscript several times.

### Conflict of interest statement

The authors declare that the research was conducted in the absence of any commercial or financial relationships that could be construed as a potential conflict of interest.

## Appendixs

### Workplace relational civility (WRC)

Annamaria Di Fabio and Alessio Gori

### Instructions

Characteristics that affect the ways of being and relating to people are shown below. The statements refer to people's interpersonal relationships at work. In the first part (A), please describe how you acted or behaved toward others (colleagues and /or superiors) over the past 3 months. In the second part (B), please describe how others (colleagues and superiors) acted or behaved toward you (in the past 3 months). Please mark with a cross all statements expressing your preference, choosing from: (1) Not at all; (2) A little; (3) Somewhat; (4) A lot; (5) A great deal. Please complete Parts A and B.

**Table:**

	**(A) Me with others**			**(B) The others with me**	
1	I was able to express my values and my beliefs calmly to others	1 2 3 4 5	1	Others were able to express their values and their beliefs calmly to me	1 2 3 4 5
2	I was able to express my point of view without being disrespectful toward others	1 2 3 4 5	2	Others were able to express their point of view without being disrespectful toward me	1 2 3 4 5
3	I respected the opinions of others	1 2 3 4 5	3	Others respected my opinions	1 2 3 4 5
4	I communicated my disagreement with others without being aggressive	1 2 3 4 5	4	Others communicated their disagreement with me without being aggressive	1 2 3 4 5
5	I was polite toward others	1 2 3 4 5	5	Others were polite toward me	1 2 3 4 5
6	I was generally kind toward others	1 2 3 4 5	6	Others were generally kind toward me	1 2 3 4 5
7	I always behaved mannerly toward others	1 2 3 4 5	7	Others always behaved mannerly toward me	1 2 3 4 5
8	I made comments that valorized others	1 2 3 4 5	8	Others made comments that valorized me	1 2 3 4 5
9	I was interested in the emotional condition of others	1 2 3 4 5	9	Others were interested in my emotional condition	1 2 3 4 5
10	I was sensitive about the difficulties of others	1 2 3 4 5	10	Others were sensitive about my difficulties	1 2 3 4 5
11	I realized the effect of my words on others	1 2 3 4 5	11	Others realized the effect of their words on me	1 2 3 4 5
12	I was attentive to the needs of others	1 2 3 4 5	12	Others was attentive to my needs	1 2 3 4 5
13	I easily recognized the feelings of others	1 2 3 4 5	13	Others easily recognized my feelings	1 2 3 4 5
